# Prognostication and optimal criteria of circumferential margin involvement for esophageal cancer after chemoradiation and esophagectomy

**DOI:** 10.3389/fonc.2023.1111998

**Published:** 2023-07-12

**Authors:** Ankit Potdar, Ke-Cheng Chen, Shuenn-Wen Kuo, Mong-Wei Lin, Hsien-Chi Liao, Pei-Ming Huang, Yi-Hsuan Lee, Hsiu-Po Wang, Ming-Lun Han, Chia-Hsien Cheng, Chih-Hung Hsu, Ta-Chen Huang, Feng-Ming Hsu, Shao-Lun Lu, Jang-Ming Lee

**Affiliations:** ^1^ Department of Gastroenterology, Global Hospital, Mumbai, India; ^2^ Division of Thoracic Surgery, Department of Surgery, National Taiwan University Hospital and National Taiwan University College of Medicine, Taipei, Taiwan; ^3^ Department of Pathology, National Taiwan University Hospital, Taipei, Taiwan; ^4^ Department of Internal Medicine, National Taiwan University Hospital, Taipei, Taiwan; ^5^ Department of Oncology, National Taiwan University Hospital, Taipei, Taiwan

**Keywords:** esophageal cancer, chemoradiotherapy, esophagectomy, circumferential radial margin, Survival

## Abstract

**Purpose:**

Circumferential radial margin (CRM) involvement by tumor after resection for esophageal cancer has been suggested as a significant prognostic factor. However, the prognostic value of CRM involvement after surgery with neoadjuvant concurrent chemoradiotherapy (CCRT) is unclear. This study aimed to evaluate the prognostic value of and survival outcomes in CRM involvement as defined by the Royal College of Pathologists (RCP) and the College of American Pathologists (CAP) for patients with esophageal cancer undergoing neoadjuvant CCRT and esophagectomy.

**Methods:**

A total of 299 patients with esophageal cancer who underwent neoadjuvant CCRT followed by esophagectomy between 2006 and 2016 were enrolled in our study. The CRM status of the specimens obtained was determined pathologically according to both the CAP and RCP criteria. Survival analyses were performed and compared according to the two criteria.

**Results:**

Positive CRM was found in 102 (34.1%) and 40 (13.3%) patients according to RCP and CAP criteria, respectively. The overall and progression-free survival rates were significantly lower in the CRM-positive group than in the CRM-negative group according to both the RCP and CAP criteria. However, under multivariate analysis, in addition to pathological T and N staging of the tumor, only CAP-defined CRM positivity was a significant prognostic factor with adjusted hazard ratios of 2.64 (1.56-4.46) and 2.25 (1.34-3.78) for overall and progression-free survival, respectively (P < 0.001).

**Conclusion:**

In patients with esophageal cancer undergoing neoadjuvant CRT followed by esophagectomy, CAP-defined CRM positivity is an independent predictor of survival. Adjuvant therapy should be offered to patients with positive CRM.

## Introduction

Esophageal cancer (EC) is a devastating disease with an increasing incidence worldwide, especially in the Western white population (1, 2). Surgery with or without radiotherapy and/or chemotherapy remains the treatment of choice for resectable EC. Nonetheless, even after en-bloc resection of the tumor, the loco-regional recurrence rates of EC are reported to be as high as 52% (3, 4). The TNM staging of EC defined by the American Joint Committee on Cancer (AJCC) is widely used for prognostication and therapeutic decision-making. In addition, various criteria have been suggested as independent prognostic factors after resection, including tumor size (5–8), tumor grade (6, 8), nodal involvement, lymph node ratio (9–13), and degree of tumor regression after neoadjuvant therapy (14).

The significance of the circumferential radial margin (CRM) status in EC has gained attention after the discovery of the association between CRM positivity and the incidence of local recurrence in colorectal and pancreatic cancer (15–17). Sagar et al. (18) first described the role of CRM in EC, showing that CRM involvement is associated with an increased risk of local recurrence. Further studies by the same group also showed that the presence of malignant cells within 1 mm of the CRM reduces median survival (19). Currently, there are two definitions of CRM involvement commonly used in clinical practice. The Royal College of Pathologists (RCP) defines a positive CRM as a tumor at or within 1 mm of the cut margin (20), whereas the College of American Pathologists (CAP) considers only the presence of a tumor at the cut margin as CRM-positive (21).

Neoadjuvant concurrent chemoradiotherapy (CCRT) before esophagectomy has been shown to improve R0 resection local control and survival compared to surgery alone (22) and is accepted as a standard of care for patients with locally advanced disease. However, the role and definition of CRM positivity after CCRT and esophagectomy remain unclear in the literature.

The purpose of the present study was to investigate the significance of CRM status in patients with EC undergoing esophagectomy after neoadjuvant CCRT and to examine the prognostic impact of CRM status according to the RCP and CAP criteria for overall and disease-free survival.

## Methods

### Patient selection and data acquisition

This study enrolled 299 patients diagnosed with EC who underwent esophagectomy after neoadjuvant CCRT at our institute between January 2006 and March 2016. The treatment plan was decided for each patient after discussion during a multidisciplinary meeting attended by the surgeon, oncologist, radiologist, physician, and nurse, according to the results of clinical staging.

Preoperative staging and routine evaluation for each patient included a computed tomography (CT) scan of the brain, neck, chest, and abdomen; upper gastrointestinogram; positron emission technology (PET) scan with CT; bronchoscopic examination; and endoscopic ultrasound (EUS). Tumor staging and grading were performed according to the 8th edition of the TNM classification of the AJCC (23).

All patients enrolled in the present study were followed up until death or five years after the initial treatment. Patient information was updated at six-monthly intervals in the first and second years after surgery and annually thereafter. Chest radiography, thoracoabdominal CT, and endoscopy were performed once or twice a year. If recurrence was suspected, the patients underwent PET/CT and endoscopic examination with biopsy.

The CRM status was analyzed separately according to criteria of the RCP and CAP from the pathological examination 1) RCP as a tumor at or within 1 mm of the cut margin (20), or 2) CAP as the presence of a tumor at the cut margin as CRM positive (21).

### Operative procedure

The procedures used for performing esophagectomy were identical to those described in our previous study (24). Patients underwent open or minimally invasive McKeown (cervical) or Ivor Lewis (intrathoracic) esophagogastrostomy depending on the location and staging of the tumor. Three-field lymph node dissections were performed, including the bilateral supraclavicular, deep cervical, recurrent laryngeal area; tracheal bronchial region; and upper, middle, and lower paraesophageal regions. Laparoscopic or open gastric mobilization and gastric tube formation, along with lymph node dissection in the hiatus, lesser curvature, left gastric artery, and celiac trifurcation, were performed. Feeding jejunostomy was performed unless the patient had already undergone the procedure prior to CCRT.

### Definitions and follow-up

Overall survival (OS) was computed as the period from the date of surgery to either the date of death or the last follow-up. Progression-free survival (PFS) was defined as the time from the date of surgery to the date of local recurrence or distant tumor relapse. The Royal College of Pathologists (RCP) defines a positive CRM as a tumor at or within 1 mm of the cut margin (20), whereas the College of American Pathologists (CAP) considers only the presence of a tumor at the cut margin as CRM-positive. Adjuvant therapy will be given if the lymph nodes were shown to have residual cancers. Post-op CCRT was the most common adjuvant therapy, which was added in the following multivariate analysis.

### Statistical analysis

Progression-free and overall survival analyses were performed using the Kaplan–Meier method. Statistical significance was assessed using the log-rank test. Hazard ratios (HRs) and confidence intervals (CIs) were obtained at 95% significance. The independent variables analyzed included age, sex, use of neoadjuvant therapy, and tumor characteristics (histology, location, length, diameter, and T stage).

The x2 test was used to assess the statistical differences between CRM involvement and other categorical clinicopathological characteristics.

The Cox regression hazard model was used for multivariate analysis to assess the independent influence of CRM status and other covariates on tumor recurrence and overall survival. Results are presented as HRs with 95% CIs. Statistical significance was set at a P < 0.05 in 2-tailed tests.

## Results

A total of 299 patients with EC were enrolled in the current study. There were 102 (34.1%) and 40 (13.3%) patients with a positive CRM according to the RCP and CAP criteria, respectively. Patient and disease characteristics are summarized in **Table 1**.

**Table 1:** The summarized patient characteristics according to RCP and CAP criteria.

Characteristic	Total N=299	RCP			CAP		
		Negative N=197	Positive N=102	P-value	Negative N=259	Positive N=40	P-value
**Age (year)**				0.775			0.120
<50	48 (16.1)	32 (16.2)	16 (15.7)		45 (17.4)	3 (7.5)	
50-65	182 (60.9)	122 (61.9)	60 (58.8)		152 (58.7)	30 (75.0)	
>65	69 (23.1)	43 (21.8)	26 (25.5)		62 (23.9)	7 (17.5)	
**Gender**				0.078			**0.054**
Female	23 (7.7)	19 (9.6)	4 (3.9)		23 (8.9)	0	
Male	276 (92.3)	178 (90.4)	98 (96.1)		236 (91.1)	40 (100)	
**pT stage**				**<0.001**			**<0.001**
pT0	93 (31.1)	93 (47.2)	0		93 (35.9)	0	
pT1	32 (10.7)	31 (15.7)	1 (1.0)		31 (12.0)	1 (2.5)	
pT2	52 (17.4)	41 (20.8)	11 (10.8)		50 (19.3)	2 (5.0)	
pT3	108 (36.1)	28 (14.2)	80 (78.4)		80 (30.9)	28 (70.0)	
pT4	14 (4.7)	4 (2.0)	10 (9.8)		5 (1.9)	9 (22.5)	
**pN stage**				**<0.001**			**0.003**
pN0	190 (63.5)	146 (74.1)	44 (43.1)		173 (66.8)	17 (42.5)	
pN1	69 (23.1)	39 (19.8)	30 (29.4)		58 (22.4)	11 (27.5)	
pN2	30 (10.0)	9 (4.6)	21 (20.6)		22 (8.5)	8 (20.0)	
pN3	10 (3.3)	3 (1.5)	7 (6.9)		6 (2.3)	4 (10.0)	
**CCRT**				**<0.001**			**<0.001**
Pre	217 (72.6)	165 (83.8)	52 (51.0)		198 (76.4)	19 (47.5)	
Pre+Post	82 (27.4)	32 (16.2)	50 (49.0)		61 (23.6)	21 (52.5)	
**COPD**				0.414			0.581
No	293 (98.0)	194 (98.5)	99 (97.1)		254 (98.1)	39 (97.5)	
Yes	6 (2.0)	3 (1.5)	3 (2.9)		5 (1.9)	1 (2.5)	
**Smoking**				0.253			0.628
No	45 (15.1)	33 (16.8)	12 (11.8)		40 (15.4)	5 (12.5)	
Yes	254 (84.9)	164 (83.2)	90 (88.2)		219 (84.6)	35 (87.5)	
**Complication**				**0.026**			0.809
No	257 (86.0)	163 (82.7)	94 (92.2)		223 (86.1)	34 (85.0)	
Yes	42 (14.0)	34 (17.3)	8 (7.8)		36 (13.9)	6 (15.0)	
RT dose*		4174.70 ± 343.37	4195.18 ± 500.71	0.972	4180.18 ± 349.50	4190.63 ± 663.50	0.684
No of dissected lymphnodes*		41.96 ± 20.23	39.81 ± 21.21	0.339	41.72 ± 20.29	38.00 ± 22.27	0.131

*Mann Whitney Test.

Bold numbers represent that they are statistically significant.

CAP, College of American Pathologists; RCP, Royal College of Pathologists; CMR, Circumferential Radial Margin; CCRT, concurrent chemoradiation; Pre OP, preoperative; Pre + Post OP, preoperatively and postoperatively.

The survival impact of each clinical and pathological variable in the univariate analysis is shown in **Table 2**. Among patients with a positive CRM as defined by the RCP and CAP criteria, significantly more patients had an advanced T and N staging status and underwent adjuvant chemoradiation than among those with a negative CRM (P < 0.05). The presence of T3 disease and lymph node metastasis increased the risk of mortality and disease progression (P = 0.001 for OS and T3 status, and P < 0.005 for other variables both in OS and PFS).

**Table 2:** The survival impact of each clinical and pathological variable in the univariate analysis.

Characteristic	Total N=299	Overall survival HR (95% CI)	P-value	Progression-free survival HR (95% CI)	P-value
Age (years)					
<50	48	1		1	
50-65	182	0.89(0.56-1.41)	0.607	0.99(0.64-1.54)	0.965
>65	69	1.06(0.62-1.80)	0.833	1.07(0.65-1.77)	0.797
Sex					
Female	23	1		1	
Male	276	1.58(0.74-3.39)	0.237	1.66(0.82-3.38)	0.163
pT stage					
pT0	93	1		1	
pT1	32	1.73(0.90-3.32)	0.098	1.81(0.99-3.31)	0.053
pT2	52	1.95(1.11-3.45)	**0.021**	2.05(1.23-3.44)	**0.006**
pT3	108	3.73(2.32-6.00)	**<0.001**	3.54(2.29-5.48)	**<0.001**
pT4	14	5.90(1.70-12.90)	**<0.001**	6.09(2.92-12.72)	**<0.001**
pN stage					
pN0	190	1		1	
pN1	69	2.13(1.45-3.14)	**<0.001**	2.29(1.59-3.30)	**<0.001**
pN2	30	3.49(2.17-5.62)	**<0.001**	2.98(1.88-4.71)	**<0.001**
pN3	10	3.05(1.40-6.67)	**0.005**	4.35(2.17-8.71)	**<0.001**
CCRT					
Pre	217	1		1	
Pre+Post	82	1.86(1.33-2.62)	**<0.001**	2.17(1.58-2.99)	**<0.001**
COPD					
No	293	1		1	
Yes	6	2.50(1.10-5.70)	**0.029**	2.11(0.93-4.80)	**0.074**
Smoking					
No	45	1		1	
Yes	254	1.32(0.80-2.16)	0.278	1.21(0.77-1.92)	0.412
Complication					
No	257	1		1	
Yes	42	0.94(0.57-1.54)	0.794	0.75(0.46-1.23)	0.256
RCP-defined CRM status					
Negative	197	1		1	
Positive	102	2.93(2.90-4.10)	**<0.001**	2.73(1.99-3.75)	**<0.001**
CAP-defined CRM status					
Negative	259	1		1	
Positive	40	4.45(2.90-6.82)	**<0.001**	2.89(2.57-5.88)	**<0.001**

Bold numbers represent that they are statistically significant.

CAP, College of American Pathologists; RCP, Royal College of Pathologists; CMR, Circumferential Radial Margin; CCRT, concurrent chemoradiation; Pre OP, preoperative; Pre + Post OP: preoperatively and postoperatively; HR, hazard ratio.

The risk of mortality and disease progression was higher in patients with CAP-defined CRM positivity, with HRs of 4.45 (2.90-6.82; P = 0.001) and 2.89 (2.57-5.88; P = 0.001) for OS and PFS, respectively. The survival disadvantage of RCP-defined CRM positivity was also significant, with HRs of 2.93 (2.90-4.10; P = 0.001) and 2.73 (1.99-3.75; P = 0.001) for OS and PFS, respectively. **Table 3** shows the multivariate analysis for patient survival.

**Table 3:** Multivariate analysis for patient survival according to the clinical and pathological variables including CRM CAP criteria.

Characteristic	Total N=299	Overall survival HR (95% CI)	P-value	Progression-free survival HR (95% CI)	P-value
Age (years)					
<50	48	1		1	
50-65	182	0.88(0.54-1.44)	0.617	1.06(0.66-1.68)	0.819
>65	69	0.98(0.55-1.75)	0.956	1.29(0.74-2.24)	0.374
Sex					
Female	23	1		1	
Male	276	1.33(0.61-2.91)	0.470	1.41(0.68-2.92)	0.360
pT stage					
pT0	93	1		1	
pT1	32	1.27(0.64-2.52)	0.504	1.31(0.69-2.47)	0.410
pT2	52	1.68(0.93-3.04)	0.088	1.60(0.93-2.47)	0.093
pT3	108	2.26(1.32-3.88)	**0.003**	2.13(1.30-3.51)	**0.003**
pT4	14	2.38(0.95-5.95)	0.064	2.16(0.90-5.18)	0.086
pN stage					
pN0	190	1		1	
pN1	69	1.86(1.19-2.89)	**0.006**	1.97(1.29-3.02)	**0.002**
pN2	30	2.55(1.49-4.36)	**0.001**	1.86(1.11-3.13)	**0.019**
pN3	10	1.89(0.80-4.47)	0.149	2.10(0.95-4.65)	0.068
CCRT					
Pre	217	1		1	
Pre+Post	82	0.88(0.59-1.32)	0.541	1.12(0.76-1.65)	0.565
CAP-defined CRM status					
Negative	197	1		1	
Positive	102	2.82(1.70-4.66)	**<0.001**	2.36(1.44-3.87)	**0.001**

Bold numbers represent that they are statistically significant.

CAP, College of American Pathologists; RCP, Royal College of Pathologists; CMR, Circumferential Radial Margin; CCRT, concurrent chemoradiation; Pre OP, preoperative; Pre + Post OP, preoperatively and postoperatively.

**Figure 1:**
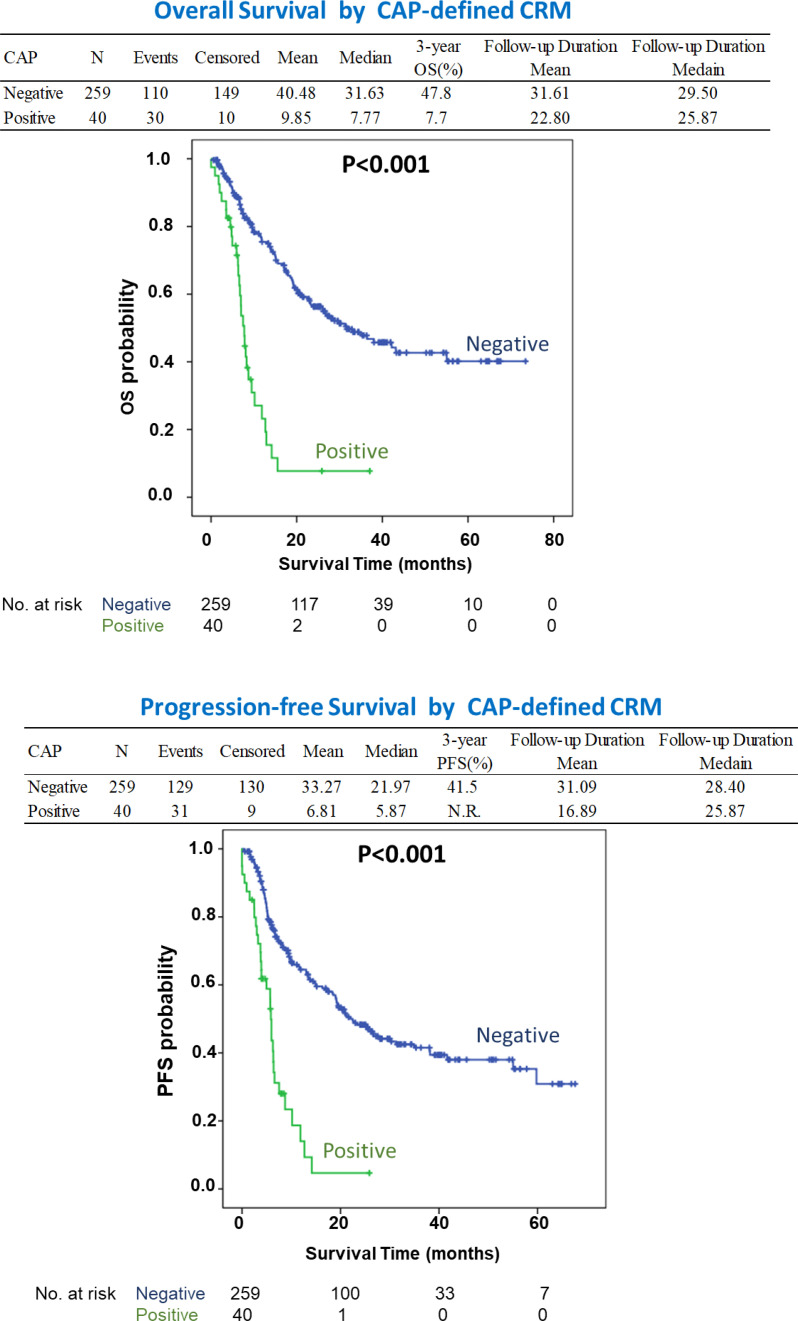
The survival curves according to CRM status based on the CAP criteria which shows patients with CRM positivity correlating with significantly lower OS and PFS (P < 0.05). NR, Not Reached.

In addition to T and N staging, the presence of a CAP-defined positive CRM strongly disadvantaged survival, with HRs of 2.82 (1.70-4.66; P < 0.001) and 2.36 (1.44-3.87; P = 0.001) for OS and PFS, respectively. When the CRM was defined by the RCP criteria, the difference became insignificant with HRs of 1.62 (0.95-2.78; P = 0.078) and 1.46 (0.89-2.40; P = 0.135) for OS and PFS, respectively (**Supplementary Table 1**). **Figure 1** shows the survival curves according to CRM status based on the CAP criteria, with CRM positivity correlating with significantly lower OS and PFS (adjusted P < 0.05).

When we further classified the patients into three groups with circumferential margin uninvolvement, less than 1 mm, and involvement the significant survival difference persisted only between the patients with and without CRM involvement, although the survival curve of patients with a clear CRM of less than 1 mm was between the above-mentioned two groups of patients. **Table 4** shows the results of the multivariate analysis, which includes the CRM status classified as CRM negativity, a clear CRM of less than 1 mm, and CRM involvement. Patients with CRM involvement showed significantly lower chances of survival, with adjusted HRs of 3.35 (1.73-6.48; P < 0.001) and 2.63 (1.42-4.86, P = 0.002) for OS and PFS respectively. This correlation was not seen in patients with a clear CRM of less than 1 mm, with adjusted HRs of 1.62 (0.95-2.78; P = 0.078) and 1.46 (0.89-2.40; P = 0.135) for OS and PFS, respectively.

**Table 4:** Multivariate analysis for patient survival according to the clinical and pathological variables including CRM with distance of margin.

Characteristic	Total N=299	Overall survival HR (95% CI)	p-value	Progression-free survival HR (95% CI)	p-value
Age(year)					
<50	48	1		1	
50-65	182	0.89(0.54-1.45)	0.640	1.06(0.66-1.69)	0.813
>65	69	0.98(0.55-1.75)	0.950	1.27(0.73-2.22)	0.393
Gender					
Female	23	1		1	
Male	276	1.36(0.62-2.96)	0.444	1.42(0.69-2.96)	0.344
pT stage					
pT0	93	1		1	
pT1	32	1.28(0.64-2.55)	0.484	1.32(0.70-2.48)	0.399
pT2	52	1.64(0.90-2.98)	0.108	1.57(0.91-2.72)	0.105
pT3	108	1.92(0.98-3.77)	**0.058**	1.93(1.05-3.55)	**0.035**
pT4	14	2.01(0.77-5.54)	0.149	1.99(0.79-5.01)	0.142
pN stage					
pN0	190	1		1	
pN1	69	1.85(1.18-2.88)	**0.007**	1.96(1.28-3.00)	**0.002**
pN2	30	2.52(1.47-4.32)	**0.001**	1.86(1.10-3.12)	**0.020**
pN3	10	1.86(0.79-4.42)	0.158	2.08(0.94-4.60)	0.072
CCRT					
Pre	217	1		1	
Pre+Post	82	0.86(0.57-1.30)	0.463	1.10(0.74-1.63)	0.648
CRM					
Uninvolved	197	1		1	
≦1mm	62	1.27(0.71-2.27)	0.418	1.17(0.69-1.99)	0.565
Involved	40	3.35(1.73-6.48)	**<0.001**	2.63(1.42-4.86)	**0.002**

CMR, Circumferential Radial Margin; CCRT, concurrent chemoradiation; Pre OP, preoperative; Pre +. Post OP, preoperatively and postoperatively.

## Discussion

Thus far, the literature has been contradictory on the significance of CRM status after esophagectomy, a fact largely attributed to the heterogeneity of the study populations (25). In addition, the different pathologic classification systems (RCP and CAP criteria) also make the prognostic effect of CRM difficult to evaluate (20, 21). Our study demonstrates that a CAP-defined positive CRM is a strong prognostic factor for patients with EC undergoing CCRT followed by radical esophagectomy and three-field lymph node dissection. However, although a similar trend was observed for RCP-defined CRM positivity in the multivariate analysis, it did not reach statistical significance.

The first study on CRM status in EC was published by Sagar et al. in 1993 (18), showing a possible association between a higher local recurrence rate and CRM involvement. In 2001, Dexter et al. (19) reported the first large-scale study on the impact of CRM involvement on the OS of 135 patients with EC. A meta-analysis by Wu et al. (26) found that the results of these studies were influenced by the heterogeneity of the patient populations, including varying T staging and the use of neoadjuvant therapy. A subgroup analysis by Khan et al. showed that CRM involvement yielded a statistically significant survival disadvantage only in T3 tumors (6). A later study by Griffiths et al. (27) revealed that the CRM status affects prognosis in patients with a low ratio of involved metastatic lymph nodes, whereas it is not a prognosticating factor in patients with a high metastatic lymph node ratio. The role of CRM status is also influenced by neoadjuvant therapy. As the above-mentioned study by Khan etal, the prognosticating significane of CRM for T3 disease was less evident once the patient received neoadjuvant chemoradiation (6). However, Shah et al. (28) reported that CRM involvement is an independent prognostic factor after deoadjuvant chemotherapy. Chao et al. (29) also reported an association between CRM status and local recurrence and survival rates in patients with ypT3 disease status after neoadjuvant CCRT.

Neoadjuvant CCRT has been adopted as a standard of care for improving the survival of patients with surgically treated locally advanced EC (22). The presence of a positive CRM after neoadjuvant therapy, especially after CCRT, represents poor response to neoadjuvant therapy and failure of complete surgical resection, leading to poor survival outcomes. However, CRM positivity, as defined by the RCP criteria, has previously been demonstrated to be 36 to 55% (18, 19, 28).

Three-field radical lymph node dissection with a mean of 41 dissected lymph nodes was performed. The association between CRM positivity and lymph node metastasis was significant in our patients. After adjusting for T and N staging status, CAP-defined CRM positivity remained a significant prognosticating factor, in contrast with RCP-defined CRM positivity. Furthermore, when patients were classified into three groups, that is, those with a clear CRM, those with a clear CRM of less than 1 mm, and those with CRM involvement, a significant survival difference was observed only between patients with and without CRM involvement. These results were compatible with the findings of Brac et al., indicating that CAP-defined CRM positivity was a significant prognostic factor for OS and PFS in patients receiving upfront esophagectomy without neoadjuvant therapy (30). Similarly, Depypere et al. reported that CAP-defined CRM positivity can precisely predict the OS and PFS in patients with ypT3 tumors after neoadjuvant CCRT and esophagectomy (31). Histologically, most of these patients had adenocarcinoma (118/163, 72.4%), and two-field lymph node dissection was performed. In contrast, all our patients had squamous cell carcinoma and underwent three-field lymph node dissection during esophagectomy following neoadjuvant CCRT.

After adjusting for other significant prognostic factors, including T and N staging, CAP-defined CRM positivity remained prognostic for the entire patient population in our study. The prognostic value of CRM involvement is, therefore, greater after radical lymph node dissection and neoadjuvant chemoradiation (32). Adjuvant therapy might therefore be prescribed on the basis of CAP-defined CRM positivity rather than the RCP criteria. What is new in our work compared to the present literature is that, in addition to pathological T and N staging of the tumor, only CAP-defined CRM positivity was a significant prognostic factor with adjusted hazard ratios of 2.64 (1.56-4.46) and 2.25 (1.34-3.78) for overall and progression-free survival, respectively (P < 0.001).

Recently, a global prospective randomized trial, CheckMate 577, demonstrated that the use of nivolumab, an adjuvant immune-checkpoint inhibitor, improves the PFS of patients with EC after neoadjuvant chemoradiation and complete esophagectomy (R0 resection) (33). It must urgently be determined whether this strategy provides a survival advantage even in CRM-positive patients, where the prognosis is poor.

This was a large cohort study conducted by a single surgical team on patients with squamous cell carcinoma after neoadjuvant chemoradiation with long-term follow-up. However, this study is limited by potential selection bias, varying surgical treatment methods, and the neoadjuvant protocol used. Further studies are required to determine whether these findings can be applied to patients with other tumor cell types, two-field lymph node dissection, and after neoadjuvant chemotherapy or immunotherapy.

## Conclusion

The CRM status, defined by CAP criteria, plays a vital role in OS and PFS in patients with EC after neoadjuvant CCRT and radical esophagectomy. Further adjuvant treatment may improve the currently poor survival outcomes of patients with CAP-defined CRM involvement after neoadjuvant CCRT and esophagectomy.

## Data availability statement

The original contributions presented in the study are included in the article/**Supplementary Material**. Further inquiries can be directed to the corresponding author.

## Ethics statement

The studies involving human participants were reviewed and approved by the Research Ethics Committee review board of the Taiwan University Hospital (202202085RINA). Written informed consent for participation was not required for this study in accordance with the national legislation and the institutional requirements.

## Author contributions

AP wrote the first draft of the manuscript. K-CC, S-WK, M-WL, H-CL, P-MH, Y-HL, H-PW, M-LH, C-HC, C-HH, T-CH, F-MH and S-LL collected data, reviewed and edited. J-ML contributed to design of the study and edit. All authors contributed to the article and approved the submitted version.

## Supplementary material

Supplementary Table 1 Multivariate analysis for patient survival according to the clinical and pathological variables including CRM RCP criteria.

Supplementary Table 2 Number of dissected lymph nodes.

Supplementary Table 3 Radiation therapy dose (cGY).
