# Supplementary material for: Prognostication and optimal criteria of circumferential margin involvement for esophageal cancer after chemoradiation and esophagectomy
Source: Front Oncol. 2023 Jul 12;13:1111998. doi: 10.3389/fonc.2023.1111998 (PMC10369182; doi:10.3389/fonc.2023.1111998)
Supplement: Supplementary Table 1 — Multivariate analysis for patient survival according to the clinical and pathological variables including CRM RCP criteria. [file Table_1.docx]

Supplementary Table 1: Multivariate analysis for patient survival according to the clinical and pathological variables including CRM RCP criteria

| **Characteristic** | **Total** | **Overall survival** | **p-value** | **Progression-free survival** | **p-value** |
| --- | --- | --- | --- | --- | --- |
|  | **N=299** | **HR (95 % CI)** |  | **HR (95 % CI)** |  |
| **Age(year)** |  |  |  |  |  |
| <50 | 48 | 1 |  | 1 |  |
| 50-65 | 182 | 1.01(0.62-1.64) | 0.968 | 1.14(0.72-1.80) | 0.591 |
| >65 | 69 | 1.12(0.63-1.99) | 0.708 | 1.26(0.72-2.18) | 0.415 |
| **Gender** |  |  |  |  |  |
| Female | 23 | 1 |  | 1 |  |
| Male | 276 | 1.54(0.71-3.34) | 0.280 | 1.61(0.77-3.33) | 0.203 |
| **pT stage** |  |  |  |  |  |
| pT0 | 93 | 1 |  | 1 |  |
| pT1 | 32 | 1.32(0.66-2.63) | 0.428 | 1.36(0.72-2.56) | 0.349 |
| pT2 | 52 | 1.55(0.85-2.83) | 0.153 | 1.54(0.89-2.67) | 0.122 |
| pT3 | 108 | 1.91(0.99-3.70) | 0.055 | 1.89(1.03-3.45) | **0.039** |
| pT4 | 14 | 3.21(1.25-8.25) | **0.016** | 2.90(1.21-6.97) | **0.018** |
| **pN stage** |  |  |  |  |  |
| pN0 | 190 | 1 |  | 1 |  |
| pN1 | 69 | 1.91(1.22-2.98) | **0.004** | 1.93(1.26-2.96) | **0.002** |
| pN2 | 30 | 2.34(1.36-4.04) | **0.002** | 1.90(1.12-3.20) | **0.017** |
| pN3 | 10 | 2.06(0.87-4.91) | 0.101 | 2.38(1.07-5.29) | **0.033** |
| **CCRT** |  |  |  |  |  |
| Pre | 217 | 1 |  | 1 |  |
| Pre+Post | 82 | 0.88(0.58-1.34) | 0.553 | 1.07(0.72-1.60) | 0.731 |
| **RCP-defined CRM status** | | |  |  |  |
| Negative | 197 | 1 |  | 1 |  |
| Positive | 102 | 1.62(0.95-2.78) | 0.078 | 1.46(0.89-2.40) | 0.135 |

RCP: Royal College of Pathologists. CMR: Circumferential Radial Margin CCRT: concurrent. Chemoradiation; Pre OP: preoperative; Pre + Post OP: preoperatively and postoperatively
