# Supplementary material for: Prognostication and optimal criteria of circumferential margin involvement for esophageal cancer after chemoradiation and esophagectomy
Source: Front Oncol. 2023 Jul 12;13:1111998. doi: 10.3389/fonc.2023.1111998 (PMC10369182; doi:10.3389/fonc.2023.1111998)
Supplement: Supplementary Table 2 — Number of dissected lymph nodes. [file Table_2.docx]

Supplementary Table 2: Number of dissected lymph nodes

|  | Q1(25%) | Median | Q3(75%) |
| --- | --- | --- | --- |
| Lymph node dissect | 25 | 39 | 52 |
|  |  |  |  |
| RCP | Q1(25%) | Median | Q3(75%) |
| Negative | 27.5 | 39 | 53 |
| Positive | 23.5 | 40 | 50 |
|  |  |  |  |
| CAP | Q1(25%) | Median | Q3(75%) |
| Negative | 26 | 41 | 53 |
| Positive | 21 | 32 | 47 |
