# Supplementary material for: Prognostication and optimal criteria of circumferential margin involvement for esophageal cancer after chemoradiation and esophagectomy
Source: Front Oncol. 2023 Jul 12;13:1111998. doi: 10.3389/fonc.2023.1111998 (PMC10369182; doi:10.3389/fonc.2023.1111998)
Supplement: Supplementary Table 3 — Radiation therapy dose (cGY). [file Table_3.docx]

Supplementary Table 3: Radiation therapy dose (cGY)

|  | Q1(25%) | Median | Q3(75%) |
| --- | --- | --- | --- |
| RT dose (cGY) | 4000 | 4000 | 4500 |
|  |  |  |  |
| **RCP** | Q1(25%) | Median | Q3(75%) |
| Negative | 4000 | 4000 | 4500 |
| Positive | 4000 | 4000 | 4500 |
|  |  |  |  |
| **CAP** | Q1(25%) | Median | Q3(75%) |
| Negative | 4000 | 4000 | 4500 |
| Positive | 4000 | 4000 | 4000 |
